# Limitations of Serological Diagnosis of Typical Cat Scratch Disease and Recommendations for the Diagnostic Procedure

**DOI:** 10.1155/2023/4222511

**Published:** 2023-03-04

**Authors:** Myrto Koutantou, Konstantinos Kambas, Sofia Makka, Pierre-Edouard Fournier, Didier Raoult, Emmanouil Angelakis

**Affiliations:** ^1^Diagnostic Department and Public Health Laboratories, Hellenic Pasteur Institute, Athens 11521, Greece; ^2^Laboratory of Molecular Genetics, Hellenic Pasteur Institute, Athens 11521, Greece; ^3^Aix Marseille Université, IRD, APHM, SSA, VITROME, IHU-Méditerranée Infection, 19–21 Boulevard Jean Moulin, Marseille 13005, France; ^4^Aix Marseille Université, IRD, APHM, MEPHI, IHU-Méditerranée Infection, 19–21 Boulevard Jean Moulin, Marseille 13005, France

## Abstract

**Introduction:**

Cat scratch disease (CSD) is the most common cause of bacterial infectious lymphadenopathy, especially in children, but its diagnosis still remains challenging. Serological assays are widely applied due to their simplicity and the non-invasive sampling. However, these techniques present several limitations, including not well-defined antigen preparation, assay conditions and cutoff titers, severe cross-reactions with other species and organisms, and the notably ranging seroprevalence in the normal population. The objective of this study is to review the literature in order to determine the best diagnostic procedure for the diagnosis of CSD.

**Methods:**

Databases including PubMed, Medline, Google Scholar, and Google were searched to determine the best diagnostic procedure for the diagnosis of CSD. A total of 437 papers were identified and screened, and after exclusion of papers that did not fulfill the including criteria, 63 papers were used.

**Results:**

It was revealed that sensitivities of serological assays varied from 10% to 100%. Indeed, more than half of the studies reported a sensitivity lower than 70%, while 71% of them had a sensitivity lower than 80%. Moreover, specificities of serological assays ranged from 15% to 100%, with 25 assays reporting a specificity lower than 90%.

**Conclusion:**

It is considered that molecular assays should be the gold standard technique for CSD confirmation, and physicians are reinforced to proceed to lymph node biopsy in suspicious CSD cases.

## 1. Introduction

*Bartonella* spp. are facultative intracellular, aerobic or microaerophilic, fastidious, and Gram-negative bacilli, and at least 13 *Bartonella* species or subspecies are known currently to potentially cause human disease. *Bartonella quintana, Bartonella bacilliformis*, and *Bartonella henselae* are responsible for most of the *Bartonella*-associated infections in humans [1]. *B. quintana* is responsible for trench fever, infectious endocarditis, bacillary angiomatosis, and lymphadenitis, *B. bacilliformis* is responsible for Carrion's disease, and *B. henselae* is responsible for lymphadenitis, infectious endocarditis, bacillary angiomatosis, and bacillary peliosis [2].

Cat scratch disease (CSD) is the most common manifestation of *B. henselae* infection which is considered the most frequent bacterial etiological agent of benign adenopathy in the young and adult population worldwide [3, 4]. However, its diagnosis has always been challenging and was originally performed using a combination of epidemiological, histological, and bacteriological criteria. The classical criteria included a cat scratch or bite, the presence of a typical CSD granuloma which consists of high numbers of B-cells and neutrophils with microabscess formation [5], negative tests for other causes of adenopathy, and a positive enzyme-linked immunosorbent assay (ELISA) or indirect fluorescent antibody (IFA) assay for *B. henselae.* Presence of three out of four criteria confirms a positive diagnosis [6].

Diagnostic techniques for CSD that are being used nowadays include culture of the pathogen, molecular techniques including polymerase chain reaction (PCR) amplification of *Bartonella* spp. genes, serological analysis [7], and detection of organisms in tissue samples by immunohistochemistry or Warthin–Starry silver staining, which has been reported to have low sensitivity but when used in combination with immunohistochemistry can offer important diagnostic value, mostly as a confirmation of the diagnosis [8]. Currently, detection of *B. henselae* DNA from lymph nodes or other clinical samples using molecular techniques is considered as the gold standard due to its high sensitivity and specificity. However, this technique is not widely used since invasive sampling, such as lymphadenectomy or biopsy, is needed [9]. Thus, serology is considered crucial in the establishment of *B. henselae* as the etiologic agent of CSD [10]. Nonetheless, immunology of CSD is still not fully understood. Although serological analysis is the most extensively studied diagnostic technique for the diagnosis of CSD, evaluations of serological tests reported variable sensitivities and specificities [11]. This is due to the high seroprevalence (reported up to ∼62%) [12] in the normal population, the significant cross-reactions that have been reported [12, 13], and several other limitations of serological assays, making it difficult to interpret the results [14].

Each serological assay suffers from limitations, resulting in the underdiagnosis of CSD. Most data about the diagnosis of CSD are based on case reports with a very limited number of subjects, and there are limited clinical studies with a standard case definition, culture confirmation, and rigidly defined disease outcomes. On the other hand, reliability of serological analysis is being questioned by many laboratories [3, 15, 16]. As a consequence, in the UK, serological testing for the diagnosis of *Bartonella* spp. infections is no longer available [17]. The objective of this article is to review current data to determine the best diagnostic procedure for the diagnosis of CSD.

## 2. Main Text

### 2.1. Search Strategy and Selection Criteria

**Figure 1:**
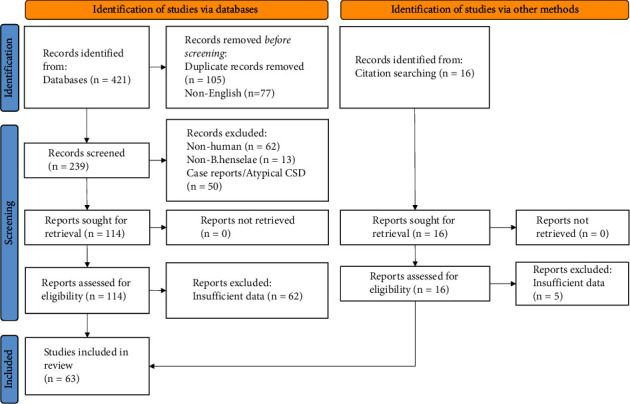
Flow diagram of study selection according to PRISMA guidelines.

In accordance with the 2020 Preferred Reporting Items for Systematic Reviews and Meta-Analyses (PRISMA) guidelines [18], a systematic search was conducted on the literature from 1950 to September of 2022 by searching PubMed, Medline, Google Scholar, and Google. We included studies related to the diagnosis of uncomplicated, typical CSD cases. Search terms included “Cat scratch disease,” “*Bartonella*,” “*Bartonella henselae*,” “diagnosis,” “serology,” “IFA,” “ELISA,” “Western Blot,” “PCR,” and “culture.” Case reports and atypical CSD-associated studies were excluded from this study, as well as articles that were not written in English and were not human-related or not *B. henselae*-related and articles in which full text was not available (Figure 1).

### 2.2. Pathogenesis of *Bartonella* spp

The infection cycle of *Bartonella* spp. is initiated by superficial inoculation of the “dermal niche” by scratching or biting, before bacteria spread to and colonize the “blood-seeding niche” which is considered to include endothelial cells [19]. From the blood-seeding niche, bacteria are periodically released into the bloodstream, where they invade, replicate, and persist within erythrocytes [20, 21]. Several animal models have been established for *Bartonella* intraerythrocytic parasitism, although the most precisely described one is a rat model for *Bartonella tribocorum* infection [22]. According to it, after inoculation, bacteria disappear from the bloodstream for ∼4 days while they replicate in the primary niche [21]. Subsequently, 5 days after inoculation, large numbers of bacteria are released into the blood circulation [22, 23]. During this time, bacteria become competent to adhere to mature erythrocytes using the Trw system [24]. After adhesion, bacteria invade and replicate intracellularly until a critical density is reached [21]. Infected erythrocytes are indistinguishable from uninfected ones and have similar lifespans, making it difficult for antibodies to function against them [22, 25]. Bacteraemia lasts for approximately 10 weeks in rats [22], whereas similar durations are observed in other experimental models of *Bartonella* infections [26].

### 2.3. Immune Responses in *B. henselae* Infection

Since in typical CSD, antibiotics offer almost no therapeutic effect and *Bartonella* is rarely isolated from patients' lymph nodes, it is assumed that the immune response plays a critical role in the development of lymphadenitis. However, there is limited information and understanding of the immune responses that take place during *B. henselae* infection or how lymphadenitis is orchestrated. Some *in vivo* and *in vitro* evidence demonstrates a Th1 polarization during *Bartonella* infection. Mouse splenocytes were able to produce INF-*γ* and IL-12 in response to *B. henselae* [27]. Moreover, high levels of circulating IL-12, IL-6, and IL-10, but absence of IL-4 and IFN-*α*, were found in patients with CSD during acute infection phase [28].

Considering that the innate immune system is usually the main sentinel against bacterial infections, very little is known about the response of innate cells against *Bartonella* infections. A study in 2006 demonstrated that dendritic cells (DCs) internalize *B. henselae*, which in turn causes DC maturation and allogeneic T-cell proliferation [5]. Subsequently, infected DCs are able to produce IL-6, TNF-*α*, and IL-10 but only minimal amounts of IL-12. Notably, they also produce CXCL13 which is the most potent chemoattractant of B-cells, suggesting an involvement in the formation of B-cell granuloma observed in CSD. Furthermore, CSD lymph node biopsies demonstrated CXCL13 positive cells surrounding B-cell granulomas. Another study showed the presence of activated macrophages, which demonstrated apoptotic phenotype, near B-cell granulomas [29]. Finally, *in vitro* experiments demonstrated that *B. henselae*-infected macrophages are not able to “digest” the bacteria since bacteria-containing vacuoles fail to fuse with acidified compartments (such as lysosomes). All the above findings indicate that the mechanisms of *B. henselae* infection and innate immune interactions are still incompletely understood and are areas where further studies are needed.

### 2.4. Local Manifestations of Typical Cat Scratch Disease

CSD is usually transmitted by the scratch or the bite of an infected cat, most commonly a kitten. It is typically caused by *B. henselae* and less commonly by *Bartonella clarridgeiae* [30], although other *Bartonella* species from other reservoir hosts like *Bartonella alsatica* [31], *B. quintana* [1], and *Bartonella grahamii* [32] have also been implicated [2, 33]. It is mostly reported in immunocompetent patients, usually younger than 20 years of age [34]. The severity of clinical manifestations of CSD highly depends on the immune status of the patient [35]. Immunocompetent patients mostly present with typical CSD that presents as a mild and self-limiting but often long-lasting swelling of the lymph nodes [1, 36]. In rare cases, CSD combines a conjunctivitis and cervical lymph nodes (Parinaud's oculoglandular syndrome) [35]. Patients with a valvular defect may develop endocarditis. However, immunocompromised patients may develop bacillary angiomatosis or peliosis hepatis [2, 37–41].

The lymphadenopathy resolves within a median of 7 weeks, with suppuration occurring in 10% to 15% of patients. However, lymph node enlargement can persist for months, with some cases exhibiting prolonged enlargement even for 12 to 24 months [1]. Patients commonly show signs of systemic infection such as malaise, headaches, weight loss, nausea and vomiting, splenomegaly, and low-grade pyrexia [1, 34, 36].

Several studies testing different antibiotic treatments have been performed, but generally antibiotic treatment of typical CSD is not recommended in uncomplicated CSD cases, as it has no significant effect on the duration of the lymphadenopathy [1, 42]. Even though the lymphadenopathy is chronic and PCR in lymph node biopsy specimens is positive for *B. henselae* DNA [3], the bacteria are identifiable with staining techniques only in the early stages of the disease. These findings, along with the fact that neither RNA is detected nor *B. henselae* is cultivated from lymph nodes, indicate that the bacterium is not alive in diseased lymph nodes and that immunology is involved in lymph node enlargement [43].

### 2.5. Molecular Assays

Molecular assays performed on pus aspirates or lymph node specimens are considered as the gold standard for the diagnosis of CSD cases based on large series of patients [3, 15]. Molecular techniques offer several advantages, including high sensitivity and specificity, rapid availability of information, and the ability to differentiate *Bartonella* organisms at the highest level [44, 45]. On the other hand, the main difficulty of these techniques is the requirement of invasive sampling [9].

Several clinical specimens have been evaluated, with lymph node pus aspirates exhibiting the highest sensitivity, followed by primary lesions, lymph node fine needle aspirations, lymph node biopsy specimens, and finally, paraffin-embedded lymph nodes [16]. Conventional PCR and real-time PCR have also been evaluated, demonstrating similar sensitivities [16].

Many DNA targets have been used to diagnose CSD by PCR with variable sensitivities. The 16S rRNA gene has been extensively studied as a potential target for CSD diagnosis, but low sensitivities have been reported [45, 46]. For example, a study of 142 real-time PCR*-*positive lymph node specimens demonstrated a sensitivity of 69% for the 16S rRNA assay [47], and another similar study of 340 lymph node specimens revealed a 56% sensitivity [48].

On the other hand, many studies tested numerous samples by real-time PCR, targeting the 16S-23S rRNA intergenic spacer region (ITS) to detect *Bartonella* species [49] along with the *pap31* gene [47] to detect specifically *B. henselae*. Indeed, using this strategy, several studies reported high sensitivities and specificities [3, 45, 47, 48]. Other targets that are being used with promising results are the *htrA* gene [15, 46, 50], *gltA*, *groEL*, *ftsZ* [51], and *ribC* [16, 52].

### 2.6. Culture

Due to its fastidious nature, *B. henselae* is very difficult to isolate, incubation lasting up to 21 days, from patients with CSD, and hence culture is not routinely recommended. *Bartonella* spp. grow on most blood-enriched media when incubated at 37°C in an atmosphere containing 5% CO_2_, with Columbia 5% sheep blood agar plates being the most commonly used. Higher recovery rates from clinical specimens have been achieved using culture in various cell lines (e.g., ECV 304 human endothelial cell monolayers) in tissue culture with a shell-vial culture technique [53]. In a study of 2,043 cases suspicious of *Bartonella* spp. infection, the recovery rate was 44% for endocarditis patients using valvular biopsy samples with the shell-vial technique instead of only 4% when culturing on blood agar [54]. Culture provides a definite diagnosis; although *B. henselae* isolation from lymph node specimens is very rare, *B. henselae* DNA can be amplified; therefore, it is suggested that *B. henselae* is not viable in diseased lymph nodes [43, 45]. Indeed, in a study of 244 PCR-positive for *Bartonella* spp. lymph node specimens, only one *B. henselae* and one *B. quintana* were successfully cultivated [3]. In another study, 340 PCR-positive for *B. henselae* lymph node specimens were cultured but only one *B. henselae* specimen was isolated [48]. Additionally, in several other studies [55], *B. henselae* could not be cultured from any lymph node specimen, as reported in a study of 80 PCR-positive lymph nodes cultured by the shell-vial technique [45], as well as from 87 PCR-positive lymph node specimens cultured on agar plates [43]. Culture of skin biopsy specimens has also been successful from three patients with CSD using the shell-vial technique [56].

### 2.7. Serology

Serology quickly became the first-line diagnostic test for CSD because of the technical challenges in isolating *B. henselae* from patient specimens and the need for invasive sampling for the detection of its DNA. Unlike culture and PCR, it does not rely on *Bartonella* being present in the specimen sample. However, the timepoint of sample analysis is important for the identification of a potential active or past infection. IgM antibodies are present for ≤3 months after exposure and IgG for up to 22–28 weeks, but 25% of the cases remain IgG-seropositive for ≥1 year according to an antibody kinetics study from 98 CSD patients [57]. Nevertheless, cases of seronegative CSD patients have also been reported [58, 59]. Additionally, it should also be taken into account that serology could be false, as in the case of intravenous immunoglobulin (IVIG) treatment, which has been reported to increase *Bartonella* IgG titers one to six days after IVIG administration [60].

IFA assay is the most widely applied method currently, for the diagnosis of CSD due to the simplicity of the method as well as the non-invasive sampling [39, 61]. It has been frequently evaluated in order to achieve a sensitive and specific diagnostic tool [2, 10]. Most of the IFA studies were conducted on series of patients lesser than 100, except one study involving 303 clinically diagnosed CSD patients [62]. Attempts to diagnose CSD have also been conducted using ELISA with studies of relatively limited series of patients, ranging from 13 [63] to 155 [64]. ELISA offers ease of use and a high level of reproducibility, but ideal antigens for use in the diagnosis of *Bartonella* infections have still not been clearly defined [44]. Studies using number of patients ranging from 7 [65] to 259 [66] have also been conducted to examine the ability of western blot (WB) to successfully diagnose CSD [67]. Apart from *Bartonella* endocarditis in which WB has demonstrated its efficiency, WB has mostly been used for research purposes and for the determination of appropriate *Bartonella* antigens for the development of a potential sensitive and specific ELISA. However, several limitations have been described in CSD serology, making result interpretation and comparison with similar studies extremely challenging.

## 3. Limitations of Serological Approaches for the Diagnosis of Cat Scratch Disease

### 3.1. Seroprevalence

**Figure 2:**
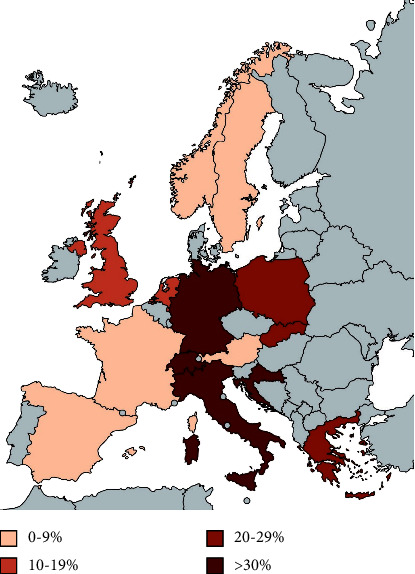
Seroprevalence of anti-*B. henselae* antibodies in European countries based on studies from 1950 to 2022.

*B. henselae* infections and CSD occur worldwide, and highly variable seroprevalence rates have been reported, preventing the correct discrimination of active and past infections. Considering the data provided in the literature, verified through serological studies, there are indications that the prevalence of infection is much higher than clinically detected [66]. Seroprevalence studies demonstrate variations in the incidence of *B. henselae* infections in many countries in Europe (Figure 2), Asia, and America. The seroprevalence rate varies greatly depending on the geographical area, the study group, and the cutoff titer, ranging from 0.1% in Norway according to a study of 1451 blood donors to almost 62% as reported in 508 healthy donors from Italy [12], making the antibody-based diagnosis of CSD problematic. Indeed, a study of 258 healthy blood donors in France showed a seroprevalence of 0.4% [68] and a similar study of 500 healthy donors from Greece revealed a seroprevalence of 20% [69]. The true incidence of CSD is difficult to be determined since it is not a reportable disease in many countries.

### 3.2. Cross-Reactivity

**Figure 3:**
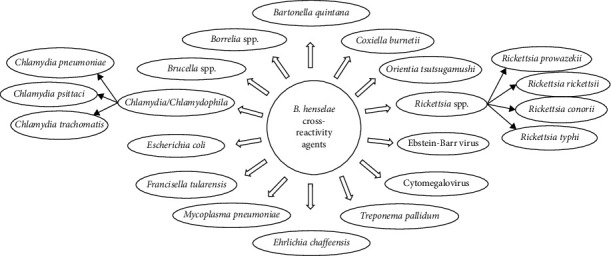
Infectious agents that cause cross-reacting serology with *B. henselae*.

Studies have repeatedly demonstrated that cross-reactivity between *B. henselae* and *B. quintana* is very high, i.e., up to 95% presumably due to close phylogenetic relatedness [11, 70, 71]. Considering that *B. henselae* and *B. quintana* can both cause lymphadenitis, infectious endocarditis, and bacillary angiomatosis [54], high cross-reactivity levels can make it difficult for serological assays to discriminate which bacterium is responsible for a specific infection. Moreover, *Chlamydia/Chlamydophila* antibodies may cross-react with *Bartonella* species [72, 73], while cross-reactivity, although at lower levels, has also been described between *Bartonella* spp. and *Coxiella burnetii* [68, 72, 74]. There are also reports of cross-reactivity to a lesser extent between *Bartonella* and *Rickettsia* spp., *Borrelia* spp., *Brucella* spp. [10], *Mycoplasma pneumoniae* [75]*, Escherichia coli, Ehrlichia chaffeensis*, *Orientia tsutsugamushi*, *Francisella tularensis*, *Treponema pallidum* [70, 73, 76], *Cytomegalovirus* [75], and Epstein–Barr virus [77] (Figure 3). In such cases, we propose a second (convalescent) sample in order to clear the situation since in most cases the antibody titers of the “true” agent rise more than those of the “false” one.

### 3.3. Antigen Preparation, Cutoff Titers, and Commercially Available Kits

Antigen preparation procedures are not well defined, resulting in significant differences in diagnostic methods between laboratories, which may impact significantly result interpretation. Several antigens are being used, including *B. henselae* strains that are agar-grown [78] or co-cultivated with mammalian cells to reduce the auto-adherent nature of the organism [77]. Moreover, whole-cell lysates are being used [64], as well as several subcellular fractions like *N*-lauroylsarcosine-soluble [79] or insoluble fractions [80] and fractions further treated with ion exchange chromatography [81, 82] or even purified recombinant proteins [63, 76, 83, 84]. Along with the different antigens, cutoff titers are not well defined either. Each laboratory uses different cutoff titers, like >1 : 64, >1 : 128, >1 : 256, or >1 : 512 for IgGs, making serological diagnosis problematic.

In addition, several commercially available kits that are widely used in CSD serologic diagnosis are described as having high sensitivities and specificities, but have been validated with inappropriate methods, such as small series of patients or cross-reaction and reproducibility controls with very limited numbers of sera. Also, significant between-kit and interpatient variability, as well as differences in analysis parameters and the subjectivity of the technician, interferes in result reading [85, 86].

### 3.4. Sensitivity and Specificity Variations

Sensitivities and specificities vary significantly among studies but remain relatively low in most cases, except from a few studies with limited series of patients [87]. Efforts to improve sensitivity usually result in significant reduction of specificity and vice versa. Consequently, many studies have displayed a lack of correlation between positive serology and PCR, and there are suggestions that serology should not be used alone but combined with other techniques such as PCR or culture to ensure accuracy in diagnostic results.

### 3.5. IFA Assay

**Figure 4:**
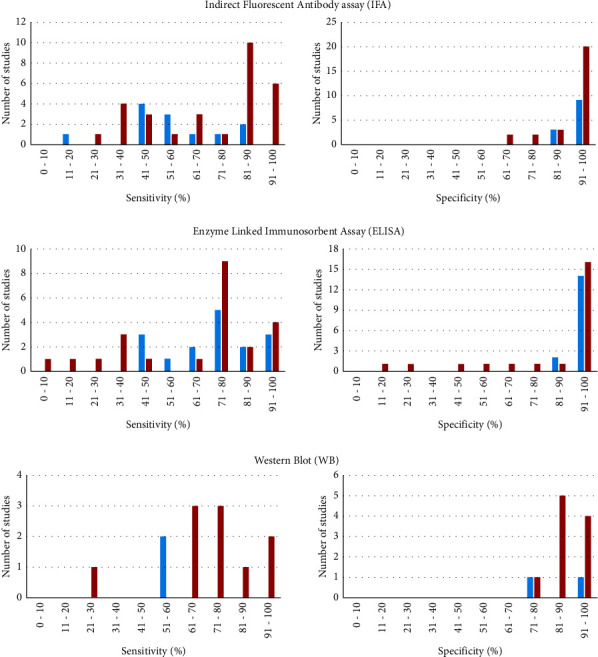
Sensitivities and specificities of serological assays provided by studies from 1950 to 2022. Red color indicates sensitivities and specificities of IgG antibody assays, and blue color indicates IgM antibody assays. Left column represents the sensitivities of the included studies, and right column represents the specificities.

Several IgM and IgG assays have been conducted, with IgM sensitivities ranging from 20% to 90% and IgG from 26% to 100%. Specificities range from 86% to 100% and 69% to 100%, respectively (Figure 4, Supplementary Table (available here)). In a study by Vermeulen et al. [85] with 50 CSD patients, several commercially available and in-house IFA assays were tested, reporting IgG sensitivities up to 98% instead of only 54% for IgM. Another study by Sander et al. [13] using two different commercial kits reported higher sensitivities for the determination of IgG titers than IgM (100% instead of 80%), but the specificity for IgG was significantly lower (70%) than that of IgM (95%).

Differences in *B. henselae* antigens between those that are agar-grown and those that are co-cultivated with mammalian cells are reported. The sensitivities from assays using agar-derived or co-cultivated antigens range from 41% to 88% and from 20% to 100%, respectively, with specificities extending from 82% to 100% and 69% to 100%, respectively. A study by Bergmans et al. [78] using 22 probable CSD cases described higher sensitivities for agar-cultivated bacteria for both IgM and IgG assays than bacteria co-cultivated in Vero cells (50% for IgM and 41% for IgG instead of 46% and 32%, respectively). However, a study by Zbinden et al. [77] published conflicting results, suggesting that Vero cell-co-cultivated *B. henselae* provided better results than agar-grown bacteria, with a sensitivity of 90% instead of 70%.

### 3.6. ELISA

Sensitivities and specificities of ELISA assays using whole-cell lysates range from 10% to 71% and from 91% to 98%, respectively. One of the ELISA studies with the largest series of patients, using 155 sera from CSD patients and 244 age-matched controls, showed specificities of 98% and IgM and IgG sensitivities of 45% and 32%, respectively. When these data were combined, the sensitivity increased to 59%. Considering the significant age-dependent increase in the IgG levels in the general population compared to CSD patients, several age-matched diagnostic models were used, resulting in a sensitivity of 64% [64].

Several recombinant proteins have been tested, with sensitivities and specificities of 43% to 100% and 15%to 98%, respectively. In a study using purified recombinant proteins as ELISA antigens, GroES, RplL, GroEL, SodB, BepA, and ABC transporter were tested. Sensitivities ranged from 78% to 86%, and specificities ranged from 15% to 59%. The rRplL ELISA was the most promising one, with a sensitivity of 78% and a specificity of 59% [84]. A study of 45 patients using r17-kDa protein as IgG ELISA antigen showed a specificity of 93% and a sensitivity of 71% [76]. The same target was used in another study of only 13 patients in an IgM assay, resulting in a sensitivity of 100% and a specificity of 97% [63]. Generally, sensitivities of IgM ELISA assays range from 45% to 100% with specificities from 86% to 99%, but these ranges increase dramatically for IgG ELISA, from 10% to 100% and from 15% to 98%, respectively (Figure 4, Supplementary Table).

Finally, several subcellular fractions have been tested, exhibiting higher sensitivities that range from 72% up to 100% and specificities of 65% to 99%. The most promising results were reported from a study using *N*-lauroylsarcosine-insoluble protein antigen, outlining a sensitivity of 96% and a specificity of 91%, but it was conducted in just 25 patients and 23 blood donors [80].

### 3.7. Western Blot

Different protein patterns in molecular weights have been described, probably due to variations in the antigen preparation methods and experiment conditions, making it difficult to compare results with similar studies [88]. Some of the WB antigens that have been studied are whole-cell lysates [67], sarcosine-soluble fraction of proteins [79], and recombinant proteins like BadA [61], 17-kDa protein [83], sucB [89], Arp [66], GroES, RplL, BepA, GroEL, SodB, and ABC transporter [84]. Additionally, significant differences have been noticed in WB patterns from patient to patient, and numerous bands have also been observed among healthy individuals as well [67]. Furthermore, inconsistent reactivity to a single band or spot by all sera has also been reported [84] (Figure 4, Supplementary Table). In an IgM-WB study of 92 suspected CSD cases, using whole-cell lysates, three immunoreactive proteins were considered relevant to CSD cases with various sensitivities (up to 53%) and a specificity of 98%, although no serum was positive for all three [67]. However, in another study of 148 patients, using recombinant Arp autotransporter protein yielded a sensitivity of 21% and a specificity of 97% [66]. Consequently, diagnostic utility of WB remains questionable and thus is currently not available as an official diagnostic test.

## 4. Conclusion

**Figure 5:**
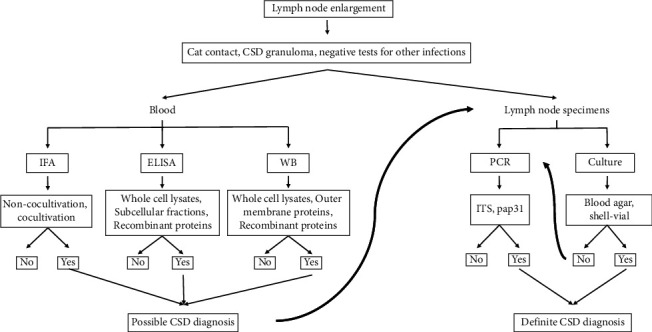
Recommendations of the diagnostic procedure of a potential CSD case.

In this review, we provide data that serological assays are highly problematic. Assay details that are not well defined, in addition to high cross-reactivity and seroprevalence, lead to variable sensitivities and specificities and inaccurate diagnoses. Considering all limitations mentioned above, suggestions have been made to stop using serological assays as reference method, but only in combination with other techniques. Indeed, in the UK, serological assays for CSD diagnosis are no longer available [17]. Many reference laboratories are considering molecular methods as the gold standard for the diagnosis of uncomplicated typical CSD from lymph node biopsy specimens [45, 47]. In patients with a cat contact, a CSD granuloma, and negative tests for other causes of lymphadenopathy, positive serological assays can only provide a possible but not a definite CSD diagnosis (Figure 5), and therefore we suggest physicians to proceed to a lymph node biopsy in case of a CSD suspicion.

## Data Availability

All data generated in this study are included in this article and the supplementary materials.

## Supplementary Materials

Supplementary Materials Supplementary table with the sensitivities and specificities of the studies used in this review is available.
