# Supplementary material for: Limitations of Serological Diagnosis of Typical Cat Scratch Disease and Recommendations for the Diagnostic Procedure
Source: Can J Infect Dis Med Microbiol. 2023 Mar 4;2023:4222511. doi: 10.1155/2023/4222511 (PMC10008113; doi:10.1155/2023/4222511)
Supplement: Supplementary Materials — Supplementary table with the sensitivities and specificities of the studies used in this review is available. [file 4222511.f1.docx]

| **Study** | **No. of patients** | **No. of controls** | **CSD confirmation method** | | | **Method tested** | **In-house/Commercial** | **Cultivation** | **Target/Antigen** | **Sensitivity** | | **Specificity** | |
| --- | --- | --- | --- | --- | --- | --- | --- | --- | --- | --- | --- | --- | --- |
|  |  |  | **PCR** | **IFA** | **Other** |  |  |  |  | **IgM** | **IgG** | **IgM** | **IgG** |
| Regnery *et al.*, Lancet, 1992. 339(8807): p. 1443-5 | 41 | 107 | - | - | Clinically suspected | IFA | In-house | Vero cocultivation | *B. henselae* whole cell | - | 88% | - | 94% |
| Barka *et al.*, J Infect Dis, 1993. 167(6): p. 1503-4 | 40 | 80 | - | - | Clinically suspected, histopathology, culture or skin test | ELISA | In-house | Agar | *B. henselae* whole cell | - | 95% | - | 99% |
| Zangwill *et al.*, N Engl J Med, 1993. 329(1): p. 8-13 | 60 (45 sera) | 112 | - | - | Lymphadenopathy, cat contact | IFA | In-house | Vero cocultivation | *B. henselae* whole cell | - | 84% | - | 96% |
| Fumarola *et al.*, New Microbiol, 1994. 17(3): p. 255-8 | 80 | 20 | - | - | Clinically suspected | IFA | In-house |  | *B. henselae* whole cell | - | 35% | - | 100% |
| Demers *et al.*, J Pediatr, 1995. 127(1): p. 23-6 | 38 | 48 | - | - | Lymphadenopathy, cat scratch | IFA | In-house | Vero cocultivation | *B. henselae* whole cell | - | 100% | - | 98% |
| Dalton *et al.*, Arch Intern Med, 1995. 155(15): p. 1670-6 | 91 strict and 375 broad criteria | - | - | - | Strict criteria, broad criteria | IFA | In-house | Vero cocultivation | *B. henselae* whole cell | - | 95% (strict) and 82% (broad) | - | - |
| Nadal *et al.*, Eur J Pediatr, 1995. 154(11): p. 906-8 | 20 | 101 | - | - | Lymphadenopathy, cat scratch | IFA | In-house | Vero cocultivation | *B. henselae* whole cell | - | 100% | - | 99% |
| Patnaik *et al.*, Clin Infect Dis, 1995. 21(4): p. 1064 | 32 | 200 | - | - | Clinically suspected | ELISA | In-house | Agar | *B. henselae* whole cell | - | 94% | - | 98% |
| Szelc-Kelly *et al.*, Pediatrics, 1995. 96(6): p. 1137-42 | 80 (56 of which with positive skin test) | 57 | - | - | Clinically suspected and 56 with positive skin test | IFA | In-house | Vero cocultivation | *B. henselae* whole cell | - | 83% (93% for those with positive skin test) | - | 98% |
|  |  |  |  |  |  | ELISA | In-house | Agar | *B. henselae* whole cell | - | 16% (18% for those with positive skin test) | - | 95% |
|  | 78 (54 of which with positive skin test) | 53 | - | - | Clinically suspected and 54 with positive skin test | ELISA | In-house | Vero cocultivation | *B. henselae* whole cell | - | 32% (35% for those with positive skin test) | - | 91% |
| Anderson *et al.*, J Clin Microbiol, 1995. 33(9): p. 2358-65 | 13 | - | - | ✓ | - | Western Blot | In-house | - | Recombinant *B. henselae* r17-kDa protein | - | 88% | - | - |
| Yoshida *et al.*, Microbiol Immunol, 1996. 40(9): p. 671-3 | 10 | 7 | - | - | Clinically suspected or histopathologically confirmed | IFA | In-house | Vero cocultivation | *B. henselae* whole cell | 20% | 50% | 100% | 100% |
| Dupon *et al.*, Scand J Infect Dis, 1996. 28(4): p. 361-6 | 64 | - | - | - | Lymphadenopathy, histopathological features of CSD | IFA | In-house | Vero cocultivation | *B. henselae* whole cell | - | 34% | - | - |
| Bergmans *et al.,* J Clin Microbiol, 1997. 35(8): p. 1931-7 | 22 | 60 | ✓ | - | Skin test, cat scratch or bite or histopathological analysis of lymph node | IFA | In-house | Agar | *B. henselae* whole cell | 50% | 41% | 100% | 95% |
|  |  |  |  |  |  | IFA | In-house | Vero cocultivation | *B. henselae* whole cell | 46% | 32% | 100% | 98% |
|  |  |  |  |  |  | ELISA | In-house | Agar | *B. henselae* whole cell | 71% | 10% | 98% | 97% |
| Zbinden *et al.*, Eur J Clin Microbiol Infect Dis, 1997. 16(9): p. 648-52 | 26 | 120 | ✓ | ✓ | - | IFA | MRL-Vero slides (MRL Diagnostics, Cypress, CA, USA) | Vero cocultivation | *B. henselae* whole cell | - | 85% | - | 93% |
| Litwin *et al.*, Am J Clin Pathol, 1997. 108(2): p. 202-9 | 131 | 10 | - | ✓ | - | ELISA | In-house | Agar | *B. henselae* OMP enriched | 94% | 86% | 99% | 96% |
| Flexman *et al.*, Med J Aust, 1997. 166(10): p. 532-5 | 303 | 102 | - | - | Lymphadenopathy (61 with cat scratch and 31 with histopathological features of CSD) | IFA | In-house | Vero cocultivation | *B. henselae* whole cell | - | 21% (62% for those with cat scratch and 90% to those with histopathological features) | - | - |
| Sander *et al.*, Clin Diagn Lab Immunol, 1998. 5(4): p. 486-90 | 20 | 270 | ✓ | - | - | IFA | BION enterprises, USA | Human larynx carcinoma cell cocultivation | *B. henselae* whole cell | 80% | 100% | 95% | 70% |
|  |  |  |  |  |  | IFA | MRL Diagnostics, Cypress, CA, USA | Vero cocultivation | *B. henselae* whole cell | 50% | 85% | 86% | 73% |
| Zbinden *et al.*, Med Microbiol Immunol, 1998. 186(4): p. 167-70 | 20 | 40 | - | ✓ | - | IFA | MRL Diagnostics, Cypress, CA, USA | Agar | *B. henselae* whole cell | 70% | - | 88% | - |
|  |  |  |  |  |  | IFA | In-house | Vero cocultivation | *B. henselae* whole cell | 90% | - | 100% | - |
| Tsuneoka *et al.*, Kansenshogaku Zasshi, 1998. 72(8): p. 801-7 | 33 | 110 | - | - | Clinically suspected | IFA | In-house | Agar | *B. henselae* whole cell | - | 49% | - | 97% |
|  |  |  |  |  |  | IFA | In-house | Vero cocultivation | *B. henselae* whole cell | - | 76% | - | 97% |
| McGill *et al.*, Infect Immun, 1998. 66(12): p. 5915-20 | 54 | 15 | - | ✓ | - | Western Blot | In-house | Agar | *B. henselae* whole cell (Bh83) | - | - | - | - |
| Not *et al.*, Acta Paediatr, 1999. 88(3): p. 284-9 | 78 | 62 | - | - | Clinically suspected | IFA | MRL Diagnostics, Cypress, CA, USA | Agar | *B. henselae* whole cell | - | 90% | - | 92% |
|  |  | 100 |  |  |  | ELISA | In-house | Agar | *B. henselae* whole cell | 80% | 75% | 98% | 97% |
| Giladi *et al.,* Clin Infect Dis, 2001. 33(11): p. 1852-8 | 84 | 220 | - | - | Lymphadenopathy, cat contact and at least one of the following (PCR, skin test or B. henselae culture) | ELISA | In-house | Agar | *B. henselae* N-lauroyl-sarcosine-insoluble outer membranes (OMP) | 48% | 75% | 100% | 98% |
| Sander *et al.*, Eur J Clin Microbiol Infect Dis, 2001. 20(6): p. 392-401 | 19 | 116 | - | - | Lymphadenopathy, negative results for other infections, negative tuberculin test | IFA | BION enterprises, USA | Cocultivated with larynx carcinoma cells | *B. henselae* whole cell | - | 37% | - | 87% |
|  |  |  |  |  |  | IFA | provided by Regnery from the CDC | Vero cocultivation | *B. henselae* whole cell | - | 26% | - | 97% |
| Maurin *et al.,* Clin Diagn Lab Immunol, 2002. 9(5): p. 1004-9 | 68 | 85 | ✓ | - | - | IFA | In-house | ECV 304 Cocultivation | *B. henselae* whole cell | - | 53% | - | 99% |
|  |  |  |  |  |  | IFA | Focus Technologies | Vero cocultivation | *B. henselae* whole cell | - | 91% | - | 87% |
| Rolain *et al.*, Clin Diagn Lab Immunol, 2003. 10(4): p. 686-91 | 52 (38 sera) | 137 (58 sera) | ✓ | - | - | IFA | Focus Technologies | Vero cocultivation | *B. henselae* whole cell | - | 87% | - | 74% |
|  |  |  |  |  |  | IFA and IFD | Focus Technologies | Vero cocultivation | *B. henselae* whole cell | - | 97% | - | 100% |
| Litwin *et al.*, J Med Microbiol, 2004. 53(Pt 12): p. 1221-1227 | 100 | 100 | - | ✓ | - | Western Blot | In-house | - | Recombinant *B. henselae* sucB protein | 55% | - | 77% | - |
| Loa *et al.*, Diagn Microbiol Infect Dis, 2006. 55(1): p. 1-7 | 45 | 86 | - | ✓ | - | ELISA | In-house | - | Recombinant *B. henselae* r17-kDa protein | - | 71% | - | 93% |
| Vermeulen *et al.*, Clin Microbiol Infect, 2007. 13(6): p. 627-34 | 51 | 56 | ✓ | - | - | IFA | In-house | Agar | *B. henselae* whole cell | 53% | 67% | 93% | 82% |
|  |  |  |  |  |  | ELISA | In-house | Agar | *B. henselae* whole cell | 65% | 28% | 91% | 91% |
| Herremans *et al.*, J Microbiol Methods, 2007. 71(2): p. 107-13 | 155 | 244 | ✓ | - | - | ELISA | In-house | Agar | *B. henselae* whole cell | 45% | 32% | 98% | 98% |
| Litwin *et al.*, Infect Immun, 2007. 75(11): p. 5255-63 | 259 | 150 | - | ✓ | - | Western Blot | In-house | - | *B. henselae* Arp protein, 2 domains | - | 21% | - | 97% |
| McCool *et al.*, Diagn Microbiol Infect Dis, 2008. 60(1): p. 17-23 | 14 | 7 | - | ✓ | - | Western Blot | In-house | Agar | *B. henselae* GroES protein | - | 71% | - | 86% |
|  |  |  |  |  |  |  |  | Agar | *B. henselae* RplL protein | - | 86% | - | 86% |
|  |  |  |  |  |  |  |  | Agar | *B. henselae* BepA protein | - | 64% | - | 100% |
|  |  |  |  |  |  |  |  | Agar | *B. henselae* GroEL protein | - | 64% | - | 100% |
|  |  |  |  |  |  |  |  | Agar | *B. henselae* SodB protein | - | 71% | - | 86% |
|  |  |  |  |  |  |  |  | Agar | *B. henselae* ABC transporter protein | - | 64% | - | 86% |
|  | 14 | 7 |  | ✓ | - | ELISA | In-house | - | Recombinant *B. henselae* rGroES protein | - | 80% | - | 15% |
|  | 18 | 17 |  |  |  |  |  |  | Recombinant *B. henselae* rRplL protein | - | 78% | - | 59% |
|  | 14 | 9 |  |  |  |  |  |  | Recombinant *B. henselae* rBepA protein | - | 86% | - | 44% |
|  | 20 | 20 |  |  |  |  |  |  | Recombinant *B. henselae* rGroEL protein | - | 80% | - | 30% |
|  | 45 | 86 |  |  |  |  |  |  | Recombinant *B. henselae* r17-kDa protein | - | 71% | - | 93% |
| Wagner *et al.*, Int J Med Microbiol, 2008. 298(7-8): p. 579-90 | 34 | 31 | - | ✓ | - | Western Blot | In-house | - | *B. henselae* BadA protein | - | 74% | - | 74% |
| Hoey *et al.*, Clin Vaccine Immunol, 2009. 16(2): p. 282-4 | 13 | 34 | - | ✓ | - | ELISA | In-house | - | Recombinant *B. henselae* r17-kDa protein | 100% | - | 97% | - |
| Herremans *et al.*, Eur J Clin Microbiol Infect Dis, 2009. 28(2): p. 147-52 | 126 | 126 | ✓ | - | - | ELISA | In-house | Agar | *B. henselae* whole cell | 56% | 36% | 98% | 97% |
| Vermeulen *et al.*, J Med Microbiol, 2010. 59(Pt 6): p. 743-745 | 50 | 55 | ✓ | - | - | IFA (Test A) – Houston-1 | Euroimmun; Fl 219b-1005M | Cocultivated Houston-1 strain with mammalian cells | *B. henselae* whole cell | 54% | - | 96% | - |
|  |  |  |  |  |  | IFA (Test B) – Marseille | Euroimmun with cocultivated Marseille strain | Cocultivated Marseille strain with mammalian cells | *B. henselae* whole cell | 50% | - | 87% | - |
|  |  |  |  |  |  | IFA (Test C) – Houston-1 | In-house | Agar | *B. henselae* whole cell | 54% | - | 93% | - |
|  |  |  |  |  |  | ELISA (Test D) – Houston-1 | In-house | Agar | *B. henselae* whole cell | 62% | - | 91% | - |
|  |  |  |  |  |  | IFA (Test E) – Houston-1 | Euroimmun; Fl 219b1005G | Cocultivated Houston-1 strain with mammalian cells | *B. henselae* whole cell | - | 88% | - | 89% |
|  |  |  |  |  |  | IFA (Test F) – Houston-1 | Focus Technologies | Vero cocultivation | *B. henselae* whole cell | - | 98% | - | 69% |
| Saisongkorh *et al.*, FEMS Microbiol Lett, 2010. 310(2): p. 158-67 | 7 | 12 | ✓ | ✓ | - | 2-D Western Blot | In-house | Agar | *B. henselae* ATPD protein | 100% | | 92% | |
| Tsuruoka *et al.*, Diagn Microbiol Infect Dis, 2012. 74(3): p. 230-5 | 46 | 88 | - | ✓ | - | ELISA | In-house | Agar | Sarcosine-soluble fraction of *B. henselae* proteins | - | 96% | - | 98% |
| Ferrara *et al.*, Lett Appl Microbiol, 2014. 59(3): p. 253-62 | 64 | 87 | - | ✓ | - | ELISA | In-house | - | Recombinant *B. henselae* r17-kDa protein | 75% | 66% | 86% | 76% |
|  |  |  |  |  |  | ELISA |  |  | Recombinant *B. henselae* rGroEL protein | 45% | 43% | 98% | 82% |
|  |  |  |  |  |  | ELISA |  |  | Combination of recombinant *B. henselae* r17-kDa and rGroEL proteins | 82% | - | 84% | - |
| Otsuyama *et al.*, J Clin Microbiol, 2016. 54(4): p. 1058-64 | 24 | 85 | ✓ | ✓ | - | ELISA | In-house | Agar | N-lauroyl-sarcosine-insoluble *B. henselae* antigen | 75% | - | 98% | - |
|  |  |  |  |  |  |  |  |  | Sarcosine-insoluble *B. henselae* antigens refined by DEAE-Sepharose Fast Flow ion-exchange chromatography | 83% | - | 98% | - |
|  |  |  |  |  |  |  |  |  | Sarcosine-soluble *B. henselae* antigens refined by DEAE-Sepharose Fast Flow ion-exchange chromatography | 75% | - | 98% | - |
| Otsuyama *et al.*, J Clin Microbiol, 2018. 56(1) | 92 | 130 | - | - | Clinically suspected, lymphadenopathy and/or cat contact | Western Blot | In-house | Agar | *B. henselae* whole cell (8 bands, not all in all samples) | 53% | - | 98% | - |
| Jost *et al.*, J Clin Microbiol, 2018. 56(12) | 10 | 16 | ✓ | - | - | ELISA | In-house | Agar | Water insoluble *B. henselae* part, fraction 24 from IEX | - | 100% | - | 93% |
|  | 33 |  | - | ✓ | - |  |  |  |  | - | 76% | - | 93% |
| Wyler *et al.*, Clin Microbiol Infect, 2020. 26(9): p. 1271-1273 | 25 | 23 | ✓ | - | - | ELISA | In-house | Agar | Sarcosine-insoluble *B. henselae* proteins | 96% | 72% | 91% | 65% |
|  |  |  |  |  |  | IFA | MRL Diagnostics, Cypress, CA, USA | Agar | *B. henselae* whole cell | 88% | 84% | 100% | 100% |
| Bayart *et al.*, Diagn Microbiol Infect Dis, 2020. 98(4): p. 115203 | 76 | 80 | - | ✓ | Clinically suspected | Chemiluminescent immunoassays | In-house | - | - | 42% | 79% | 98% | 94% |
